# Skin and Mechanoreceptor Contribution to Tactile Input for Perception: A Review of Simulation Models

**DOI:** 10.3389/fnhum.2022.862344

**Published:** 2022-06-02

**Authors:** Davide Deflorio, Massimiliano Di Luca, Alan M. Wing

**Affiliations:** SyMoN Lab, School of Psychology, University of Birmingham, Birmingham, United Kingdom

**Keywords:** simulation models, tactile neuron, mechanoreceptor, glabrous skin, tactile perception, neurophysiology, advancements and challenges

## Abstract

We review four current computational models that simulate the response of mechanoreceptors in the glabrous skin to tactile stimulation. The aim is to inform researchers in psychology, sensorimotor science and robotics who may want to implement this type of quantitative model in their research. This approach proves relevant to understanding of the interaction between skin response and neural activity as it avoids some of the limitations of traditional measurement methods of tribology, for the skin, and neurophysiology, for tactile neurons. The main advantage is to afford new ways of looking at the combined effects of skin properties on the activity of a population of tactile neurons, and to examine different forms of coding by tactile neurons. Here, we provide an overview of selected models from stimulus application to neuronal spiking response, including their evaluation in terms of existing data, and their applicability in relation to human tactile perception.

## Introduction

The sense of touch is fundamental to our everyday life. It enables us to discriminate material properties, to identify objects, and to act on and interact with the external world, including affective and social exchange. It is the first sense to develop in the womb, at 8 weeks embryos respond to tactile stimulation (Bradley and Mistretta, 1975), and works *via* the largest organ of the body, the skin. The lack of tactile perception undermines safe and successful interaction with the environment and to some extent impacts independent living as is the case with people affected by peripheral neuropathy (Cole, 2016).

Understanding how touch sensory signals arise at the periphery and are processed at the central level is important for research and applications in many fields, such as neuroprosthetics and neurorehabilitation, service robots to assist the elderly or robotics applied to industry, and haptic devices to assist surgery or visually impaired people. For example, implementing biomimetic sensory feedback based on the known properties of mechanoreceptors and the way in which tactile features are extracted (e.g., spike timing versus mean firing rate) has helped to improve the quality of neural prostheses in delivering tactile sensations (George et al., 2019).

There are several issues concerning research on the sense of touch ranging from the peripheral acquisition of sensory information to the transformation of this signal into a meaningful percept. Here, we consider two main questions related to the early stages of sensory processing. The first is how do the mechanical properties of the skin affect the activation of the receptive organs? The second is what are the relevant features of the signal sent to the central nervous system by the large number of mechanoreceptors that work together to create the sense of touch? The skin is composed of different layers that have complex, non-linear mechanical properties. As a consequence, it is difficult to characterise and predict how skin stretches and deforms under different stimulations. Tactile signals are also complex and highly variable as they are generated by a large number of mechanoreceptors, hundreds in the finger pad alone, which respond to mechanical deformation in a variety of ways.

Skin behaviour and mechanoreceptors response are intertwined and should be concurrently addressed when investigating the sensory mechanisms of tactile perception. Yet, because of their complexity, it can be especially challenging to study them together, and various simplifying assumptions have been adopted to allow this.

A common approach to investigate the mechanics of the skin is to use video recordings, displacement sensors such as vibrometers, accelerometers, ultrasound scanners, or suction and indentation devices (Diridollou et al., 2001; Delhaye et al., 2016; Greenspon et al., 2020), to measure the stresses and strains at the level of the skin. However, in practice, it is difficult to use these methods while recording the activity of a population of tactile neurons with microneurography, which allows direct observation of peripheral nerve activity *in vivo* with a high temporal resolution. These recordings are even more challenging when combined with the constraints of psychophysical testing.

Microneurography recordings are performed by inserting a very fine needle electrode through the skin and into an underlying nerve fibre to register the potential across the afferent fibre membrane. The experimenter has to place the electrodes by hand through a process of trial and error (typically listening to the electrical discharge pattern signifying the needle tip has penetrated a nerve fibre) which makes the task difficult and requires fine manual dexterity and takes a lot of time and patience. In addition, only a single fibre or a small number of fibres can be recorded at a time. Thus, recording the response of a high number of fibres would require multiple sessions on the same task.

Notwithstanding these limitations, animal and human studies have provided enough knowledge for the development of computational models to predict skin behaviour under specific circumstances (e.g., Pawluk and Howe, 1999) as well as to simulate the activity of tactile neurons in response to a variety of stimuli (e.g., Sripati et al., 2006). Having functional quantitative models that can reproduce the behaviour of the skin and the response of tactile neurons is helping to overcome the limitations of classical recording techniques and address the two questions mentioned above. In particular, models allow the running of computer simulations that can be a viable way to study the relation between skin properties and neuron population response and to assess differences in tactile neural coding. However, it is important to keep in mind that this approach comes with limitations and still requires validation with real data, especially when the simulations are performed on stimuli that differ from the ones used to build the original model.

The aim of this paper is to inform researchers in psychology, sensorimotor science and robotics who seek to implement quantitative models in their research. The focus is: (i) to provide an overview of the available models of the transduction process from stimulus application to neuronal spiking response; (ii) to evaluate the models in relation to existing data; and (iii) to determine their applicability in relation to human tactile perception. In the first section, we outline the most important properties of the glabrous skin of the finger and the skin behaviour that make modelling touch difficult (i.e., complex structure and viscoelastic properties). In the second section, we summarise how the mechanoreceptors work. Then, we provide a summary of what is currently understood about the link between peripheral activity (i.e., skin and mechanoreceptors) and human tactile perception, to highlight why it is important to study the population activity of tactile fibres. Finally, we present a selection of models that simulate the activity of tactile fibres and describe the advantages, limitations, and applications of each model. We do not consider models that address the processing of tactile information at the cerebral cortex or decision-making processes but rather the characteristics of the information that is sent to the brain.

## Overview of the Biomechanics-Related Properties of Glabrous Skin

The glabrous skin exhibits complex mechanical behaviours which increase the difficulty in the development of realistic models. Rather than making an exhaustive description of the biological origins of the mechanical properties of the skin, here we intend to provide a summary of the most prominent requirements that a biomechanical model should consider. Our goal is to highlight that the theoretical and computational requirements need to be carefully considered before undertaking a quantitative modelling effort.

### Skin Composition and Properties

There are important differences in skin properties, sensory abilities, and perceptual significance across the body. The major one is between hairy skin (e.g., on the back of the hand and forearm) and non-hairy, glabrous skin (e.g., on the finger pads, palm and soles of the feet). Hairy skin, amongst other functions, is important for affective touch which is mediated by slow-conductive, unmyelinated C-tactile afferents (McGlone et al., 2007). Glabrous skin, instead, is considerably more sensitive in discriminative judgement thanks to its morphology and the presence of very large numbers of mechanoreceptors innervating fast-conductive, myelinated A-tactile fibres.

**FIGURE 1:**
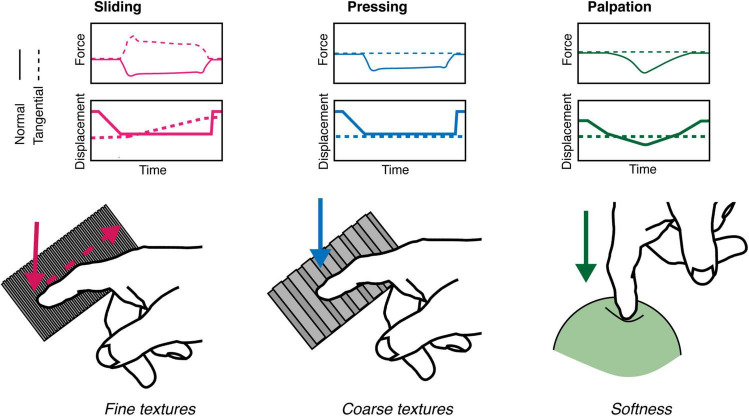
Schematic view of exploratory movements. Surface texture (e.g., periodicity of a spatial grating, or roughness of sandpaper) may be felt by static pressing or sliding contact of the index finger with the normal and tangential force components as shown. Sliding is critical in discriminating very fine texture as it generate skin vibrations which reflect the sensed surface.

In classifying objects on a range of different properties, such as texture, hardness or shape, we employ specific exploratory movements with the digits that are optimal for extracting cues relevant to those properties (Lederman and Klatzky, 1987). The contact (e.g. sliding vs pressing) between the glabrous skin and the properties of the touched object (e.g., fine or coarse texture and compliance, Figure 1) and the physical details of the interaction (e.g., normal and tangential force and displacement) determine how the skin deforms and the mechanoreceptors are activated, and in turn how the stimulus features are coded by the activity of sensory neurons (Bensmaia and Hollins, 2003; Muniak et al., 2007; Weber et al., 2013; Greenspon et al., 2020). However, the relationship between specific stimuli and the resulting spatiotemporal deformation of the skin is not straightforward but, in fact, is quite non-linear. This is because the skin is a highly complex medium composed of multiple layers having different load- and time-dependent properties (Daly, 1982; Wang and Hayward, 2007). These comprise epidermis, dermis, and subcutaneous tissue (hypodermis), which is not part of the dermis but is important for providing attachment of the skin to the bones and muscle.

**FIGURE 2:**
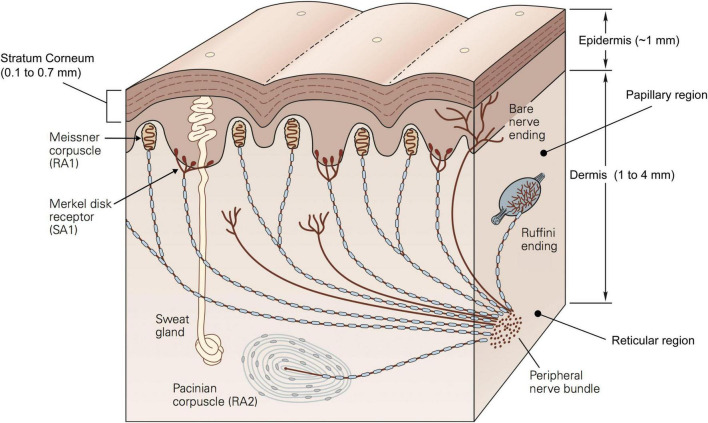
Schematic representation of a cross section of the glabrous skin showing different layers, mechanoreceptors and other components. Republished with permission of McGraw Hill LLC, from Kandel et al. (2013) 5/E, permission conveyed through Copyright Clearance Center, Inc.

The epidermis is the outer skin layer and is further subdivided into multiple layers, of which the stratum corneum is the outermost one contacting the external world. The thickness of the stratum corneum is highly variable across individuals ranging between 0.1 and 0.7 mm on the finger pad (Fruhstorfer et al., 2000). This property is relevant as it might indirectly contribute to skin friction by affecting the distensibility of the skin when sliding (Liu et al., 2015). The composition and higher thickness of the dermis (1 to 4 mm) make it functionally more important than the stratum corneum. The dermis hosts most of the mechanoreceptors and nerve endings involved in conveying information about touch and temperature (Figure 2). It contains sweat glands and sebaceous glands which contribute to the hydration of the skin which affect its frictional properties, the contact dynamics, and in turn the way we interact with objects (André et al., 2010; Adams et al., 2013).

The dermis can be histologically divided into two regions: the papillary region and the reticular region. The papillary region consists of loose connective tissue with fingerprint-like projections called papillae which extend to the epidermis and form the papillary ridges of the fingerprint. These irregularities contribute to how the skin responds to mechanical stimuli, mainly by affecting the contact area, and so the friction between the finger and the object (Duvefelt et al., 2016; Arvidsson et al., 2017), as well as the distribution of stress fields (Gerling and Thomas, 2005). On the other hand, the reticular region constitutes the bulk of the dermis and acts as the support structure. As such, it constrains the deformation that the dermis can undergo. The connective tissue of which the reticular region is made, is very dense and incorporates both collagen fibres, which have high tensile strength and form the main supporting structure, and elastomeric fibres, which are elastic and thus enable the skin to return to its original shape following deformation. Together the two sets of fibres generate directionally specific mechanical properties as well as viscoelastic qualities (Daly, 1982). In fact, the skin behaves differently when subject to stresses and strains along or across the finger. Such anisotropy is reflected by the higher tension of the skin across the Langer’s lines (Langer, 1861). Langer discovered these lines when he observed that a circular incision on a corpse changed into an oval shape. These tension lines may be due to the orientation of the collagen fibres which are parallel to them (Gibson et al., 1969).

Viscoelastic materials exhibit stress/strain characteristics that lead to time-dependent non-linear behaviours. These behaviours include stress relaxation, hysteresis, and creep which can last several seconds. Stress relaxation is measured by stretching and holding the skin and measuring the force required to hold the skin at a given distance. At low strains, the skin responds as an elastic body so that the force required is the same over a long time-interval. At high strains, it behaves as a viscoelastic body requiring a force decreasing logarithmically with time (Daly, 1982; Pan et al., 1997). In addition, the stress-strain relationship is further characterised by a non-linear response. Under low uni-axial loading the skin is relatively soft but it gets stiffer for high loads for both normal (Maurel et al., 1998) and tangential displacement (Nakazawa et al., 2000). Hysteresis is defined as the strain energy loss between loading and unloading phases due to internal friction, that is the skin deformation decreases during unloading more slowly than it increases during loading. The energy loss is high, repeatable, and invariant for long loading-unloading cycles (e.g., 20–80 s), but it diminishes for faster cycles (e.g., 5 or 10 s) (Wang and Hayward, 2007). Mechanical creep is the continuous extension of the skin under a constant force. It has been shown that skin creep can be divided in three parts: an initial purely elastic deformation, a viscoelastic phase, and a constant creep phase (Agache, 2000). This phenomenon begins with the realignment of the collagen fibres that are rearranged in parallel to one another when stretched (inherent extensibility). During stretching, water in the collagen network is displaced, and elastic fibres are micro-fragmented resulting in mechanical creep and a more viscous skin (Wilhelmi et al., 1998).

Skin properties can be highly variable across different individuals. For example, changes in skin elasticity correlate with age, gender (Yang et al., 2018), occupation, and history of exposure to environmental factors such as the sun (Langton et al., 2017). In particular, with ageing, the elastomeric proteins become sparser and their orientation changes resulting in less elastic skin (Yang et al., 2018). In a modelling study it has been shown that the lower elastic modulus observed in the elderly affects the distribution of stresses and strains during static indentation and lateral sliding. As a result, skin vibrations that are generated by sliding the finger across a surface are higher in amplitude and frequency range which may negatively influence the response of the mechanoreceptors (Amaied et al., 2015).

Skin properties are also affected by factors in the testing environment. Although skin hydration is regulated by the glands located in the dermis, it is highly susceptible to environmental conditions such as temperature and humidity, the application of water, or other formulations. For example, Sandford et al. (2013) measured the skin stiffness of the arm when exposed to different levels of relative humidity at constant temperature. They found that skin hydration increases with relative humidity and that skin stiffness has a positive relation with the hydration level. This is relevant because skin hydration is also positively correlated with friction (André et al., 2010) which affects vibration in sliding and contributes to how the tactile receptors are activated.

### Modelling the Skin

The complexity and variability of skin biomechanics poses a challenge to the development of realistic and computationally efficient models. One way to capture the deformation and reproduce the behaviour of the skin is to employ continuum mechanics and finite element techniques. Continuum mechanical modelling involves a simplified characterisation of the skin to predict its deformation and simulate the mechanoreceptors response properties accurately. The skin is often assumed to be homogenous, isotropic and linearly elastic. On the other hand, finite element modelling aims to provide a more realistic description of the different layers of the skin having different thickness and mechanical properties (i.e., viscoelasticity) as well as being influenced by adjacent bones and nails. Typically, finite element modelling is focused more on quantifying the exact relationship between specific loads applied to the skin and the resulting deformation, and less on the neural response. However, finite element modelling can be demanding as it requires the construction of a 2D or 3D mesh of interacting elements each with a set of parameters based on actual measurements of the finger. Thus, choosing one approach or the other will depend on the scope of the model. For example, applications in neuroprosthetics and robotics require fast computations to be carried out in real-time. As such, a simplification of the skin mechanics may be advantageous in this kind of scenario.

A further issue is related to the transformation of the skin response into neural activity. A common approach is to derive the stresses and strains resulting from contact with a specific stimulus at the level of the receptor of interest and to transform them into spike trains and/or firing rates. The question is then which of the several measures of stress and strain that have been successfully tested is preferable, including strain energy density, maximum compressive stress and strain, von Mises stress, change in the receptor area, and combinations of these measures (Phillips and Johnson, 1981b; Connor and Johnson, 1992; Dandekar et al., 2003; Sripati et al., 2006: Gerling et al., 2013). In addition, there are other mechanical cues that can be exploited by the peripheral nervous system. For example, the strain fluctuation variation, defined as the mean absolute difference of the maximum compressive strain between pairs of sample points in the skin, has been recently used to successfully predict the perceptual roughness of 3D printed objects (Tymms et al., 2018). Similarly, the variations in tangential force generated when sliding the finger on a regular texture reflects the interaction between the skin and the geometry of the surface and has been used to predict the subjective estimation of the surface roughness (Smith et al., 2002), or the performance in a roughness discrimination task (Roberts et al., 2020).

## Tactile Receptors of the Hand

Regardless of the specific skin deformation that is at the origin of the simulated spiking activity, it is important that the model can reproduce the properties of each afferent type and take into account the neural response at the population level. In this and the following section, we outline the most relevant response properties and provide evidence for the need to study the response of multiple afferents.

**FIGURE 3:**
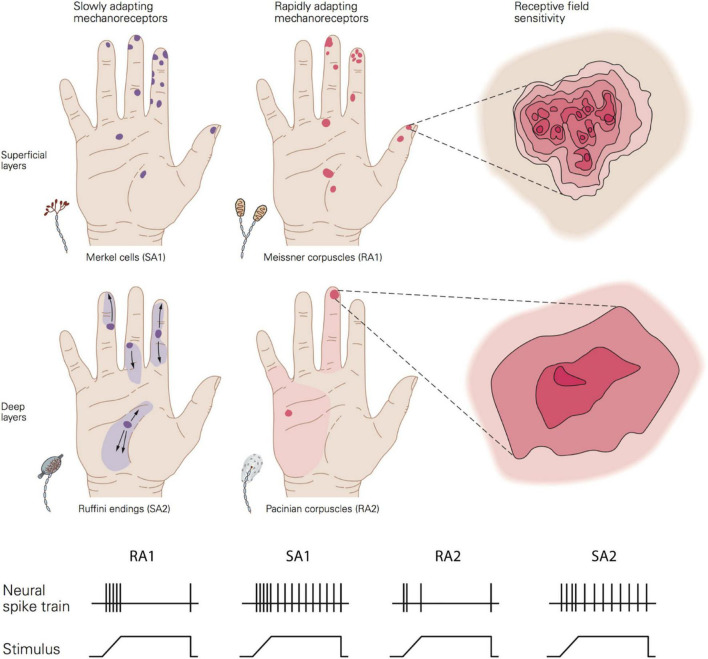
Top image shows a schematic view of the distribution, size, and sensitivity map of the receptive field of the four afferent types. Bottom image show the typical adaptation of the four afferent types in response to a ramp-and-hold indentation. Republished with permission of McGraw Hill LLC, from Kandel et al. (2013) 5/E; permission conveyed through Copyright Clearance Center, Inc.

Tactile perception is mediated by mechanoreceptors located in the dermis that are sensitive to skin mechanical deformation. They are connected to type II A fibres which transmit the information to synapses in the dorsal spinal cord, through the thalamus and then to somatosensory cortex for central processing. There are four types of mechanoreceptors that differ from one another in terms of their morphology, distribution, and response properties (Figure 3). These properties include: (i) adaptation or the rate at which the neural response subsides to a constant static stimulus (slow and rapidly adapting receptors); (ii) the receptive field characteristics; (iii) frequency sensitivity profile to vibratory stimuli (e.g., sinusoid) which may be defined in terms of absolute threshold (the minimum amplitude that elicits a spike for a specific frequency) and tuning threshold (the minimum amplitude that elicits at least one spike per cycle); (iv) the spike timing, or the precise and repeatable occurrence of individual spikes.

Adaptation rate and receptive field properties are commonly used to categorise the type of receptors. Meissner corpuscles are referred to as rapidly adapting type 1 receptors (RA1). Located close to the skin surface at the base of the epidermis (0.5–1 mm below skin surface), they respond preferentially to changes in applied load in a frequency range from 1 to 300 Hz (most sensitive between 5 and 50 Hz) (Bolanowski et al., 1988; Kandel et al., 2013). Their response subsides quickly to static deformation of the skin. Pacinian corpuscles are rapidly adapting type 2 receptors (RA2 or PC). This type of receptor can be found at a deeper layer of the skin, in the dermis (2–3 mm), and they respond to changes in applied pressure for a wide range of frequencies from 5 to 800 Hz (most sensitive between 30 and 500 Hz) (Bolanowski et al., 1988; Kandel et al., 2013). Similar to RA1, their response fades out rapidly to sustained indentation.

Merkel cells constitute slowly adapting type 1 units (SA1). Located close to the skin surface at the tip of the epidermal sweat ridges (0.5–1 mm below the skin surface). They respond to the dynamic onset and the subsequent sustained static pressure with slow adaptation to frequencies up to 100 Hz (most sensitive between 0.3 and 5 Hz) (Bolanowski et al., 1988; Kandel et al., 2013). Finally, Ruffini endings are mainly located around the nail bed and rarely found in the deeper layer of the dermis (2–3 mm). Ruffini endings respond to stretching during both the dynamic and static phase of skin indentations. They were classically thought to be connected to SA2 fibres, but more recent evidence suggests that only a very few SA2 fibres are likely to innervate Ruffini endings (Parè et al., 2003).

It has been estimated that human adult skin has around 200.000 and 270.000 neural fibres linked to mechanoreceptors (Corniani and Saal, 2020), but their density varies across the body with a higher concentration in the hand, feet, and face. The glabrous skin of the young adult hand alone has 17,000, which are more prevalent on the finger pad (Johansson and Vallbo, 1979). The number of Meissner and Merkel’s cells decrease with ageing, with the age group 60–90 years old having four to six times fewer receptors than younger adults between 20 and 49 years of age (García-Piqueras et al., 2019). The morphology of mechanoreceptors also changes with ageing. For example, Meissner’s corpuscles become smaller and denervated. These receptor changes may contribute to the deterioration of tactile spatial sensitivity observed with ageing.

The area of the skin to which each fibre can respond to is termed the receptive field. Type 1 fibre (i.e., SA1 and RA1) refers to small receptive fields while type 2 fibre (i.e., SA2 and RA2 or PC) refers to large receptive fields. This property depends in part on the depth of the mechanoreceptors with superficial receptors (i.e., SA1 and RA1) having small receptive fields and deeper receptors (i.e., SA2 and RA2 or PC) large receptive fields.

The size of receptive fields is also correlated with the density of receptors and the sensitivity of a specific body part. Thus, the higher density of receptors at the finger pad and the smaller receptive fields lead to higher sensitivity than, for example, the palm of the hand where receptors are more sparsely distributed (Figure 3). In the finger pad there are about 100 SA1 units and 150 RA1 units per cm^2^ corresponding to an average centre to centre spacing of their receptive fields of 1 and 0.82 mm, respectively. Importantly, the receptive field is a functional concept which depends on the stimulus parameters, as first noted by Johansson (1978). In fact, it has been shown that the receptive field area of SA1 and RA1 fibres increase linearly as the indentation depth increases with estimated minimum area of 5 mm^2^ for both and median areas of 11 mm^2^ for SA1 and 12.6 mm^2^ for RA1 (Vega-Bermudez and Johnson, 1999). Although the afferent spacing and receptive field size is related to and might limit the tactile spatial sensitivity of a specific body area, these are not the only factors involved. In particular, RA1 and SA1 fibres innervate multiple receptor organs and, at the same time, each receptor is linked to multiple fibres. An RA1 fibre innervates 10 to 30 Meissner corpuscles on average and each Meissner corpuscle is innervated by 2 to 5 RA1 fibres. This innervation branching allows the combination of information from several adjacent regions of the skin and contributes to the receptive field properties of type 1 units. Having multiple “hotspots” may benefit the perception of fine spatial features such as the detection of small changes in edge orientation, important for fine manual dexterity (Pruszynski et al., 2018). All these properties enable RA1 and SA1 receptors and associated fibres to transmit detailed spatial representations of an object’s geometry when in contact with the skin.

In contrast with RA1 and SA1, Pacinian corpuscles (RA2) are more sparsely distributed (Johansson and Vallbo, 1979), and only a single afferent fibre is connected to each Pacinian receptor. Their receptive fields are relatively large (5–10 times that of SA1) (Johansson and Vallbo, 1980) which makes these fibres unsuitable for resolving fine spatial details. Nonetheless, the high sensitivity of RA2 to sub-micron vibrations of the skin, over a wide range of high frequencies, plays a major role in the detection of very fine features during dynamic exploration. For example, LaMotte and Srinivasan (1991) found that RA2 fibres responded consistently during a sliding movement over a texture composed of bars with a height of only 0.05 μm. They showed that these values are in agreement with human detection thresholds suggesting that RA2 units might play a major role for detecting this sort of micro feature. SA2 fibres, instead, tend to be distributed near the fingernails, which makes them less sensitive than RA1 and SA1 to the transient components of sensations, and it has been shown that they might contribute to the perception of applied force (Brothers and Hollins, 2014). However, the role of Ruffini receptors is still not fully determined, as most neurophysiological studies on touch have been conducted on monkeys’ glabrous skin rather than humans’, which is devoid of SA2 fibres.

## Tactile Perception

Humans are able to detect and discriminate many different classes of stimuli. Such sensitivity is due to the ability to code spatiotemporal patterns of the stimulation by leveraging the properties of individual receptors, combining skin pressure information close to the indentation site (static component) with the variations of pressure (i.e., vibrations/waves) that propagate away from the contact points (dynamic component) throughout the hand up to the wrist (Shao et al., 2016). Interestingly, the stimulation originating from long-distance propagation of waves to remote receptors can alone still be used for sensory discrimination in some instances. For example, patients with pathological denervation of the fingertip and participants with an anaesthetised finger are unable to do tasks based on local information (like grating orientation discrimination using static touch). However, by using only information like vibration propagated to the wrist during sliding touch they can perform roughness discrimination tasks (Libouton et al., 2012). The receptors involved in this task are thought to be primarily Pacinian Corpuscles (RA2) which are located in the deeper layers of the skin as well as in the proximity of muscles, tendons and ligaments (Mountcastle, 2005). However, it has been observed that proprioceptive receptors such as Golgi tendon organs and muscle spindle primary and secondary endings are also sensitive to small vibrations (Fallon and Macefield, 2007). In particular, Golgi tendon organs seem to respond to vibrations during voluntary muscle contraction but not when the muscles are relaxed suggesting that this type of mechanoreceptor may contribute to tactile sensation during active movement. Interestingly, this long-distance activation seems to be driven by Rayleigh waves that can propagate through all the skin layers equally without any energy loss and are not affected by the stiffness of the stratum corneum, unlike pressure waves and shear waves (Andrews et al., 2020).

### Spatial and Temporal Code

The contrasting findings for static and sliding touch suggest that tactile perception relies on at least two different neural mechanisms. In fact, the encoding of tactile information may involve a spatial or a temporal code depending on the circumstances. The former refers to the firing rate variations between afferents due to the layout of the pattern indented into the skin. The latter, in contrast, employs firing rates variations of individual afferents over time. Models should consider these two components depending on the type of stimuli involved.

**FIGURE 4:**
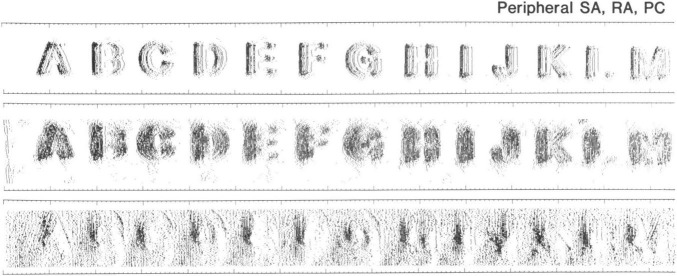
Somatosensory Evoked Potentials (SEP) in rhesus monkeys for several indented letters. Rows from top to bottom show SEPs from one SA1, one RA1, and one RA2, respectively. Image reproduced with the author’s permission (Phillips et al., 1988).

In a seminal work, Phillips et al. (1988) showed that during static contact, the spatial layout of the indentation is reflected in the spatial activation of SA1, and to a lesser extent, RA1 fibres. They repeatedly indented each letter of the alphabet into the finger of rhesus monkeys while shifting the position of the letter on each iteration. Then, they plotted the generated action potentials of the 89 recorded afferents (34 SA1, 36 RA1, and 19 RA2) to build a Spatial Event Plot (SEP). These plots showed that SA1 responses carry fine spatial details of the image, while RA1 plot are less sharp, and RA2 plots even more blurred (Figure 4). In fact, SA1 and RA1 have small receptive fields and are present at high density which make them suited to resolve fine spatial details. However, it is not clear whether the innervation density limits tactile acuity or whether there are factors that allow perception of features with a resolution beyond the spacing of afferents.

Afferent spacing was initially considered as a limit to the resolution of fine details by static touch, but several studies showed that hyperacuity (Westheimer, 1975) is present in the tactile as well as in other sensory modalities. For example, it has been shown that edge orientation thresholds are around 20° in a 2-IFC discrimination task (Bensmaia et al., 2008a). The authors suggested that tactile discrimination of bars is poor when compared to the visual counterpart, but they are similar in the extent to which the resolution limit is set by the respective innervation density of both modalities. However, in a recent study using a tactile pointer-alignment task, Pruszynski et al. (2018) showed that participants can detect very small changes in the orientation of edges (i.e., 3° for edges spanning the entire fingertip – 10 mm). These findings are in contrast with the traditional view that tactile spatial resolution is limited by the afferent density and the centre-to-centre spacing of the receptive fields (Dodson et al., 1998; Friedman et al., 2002). Pruszynski et al. (2018) used a modelling approach to show that implementing complex receptive fields with multiple hotspots of high sensitivity can enable the tactile system to resolve fine details near the limit of receptive field spacing. These results suggest that tactile orientation discrimination can be as good as vision. Recently, Jarocka et al. (2021) provided further evidence that the spatial sensitivity of SA1 and RA1 is defined by the complex structure of their receptive fields having high responsive subfields and not only their spacing. They estimated that the subfield acuity is approximately 0.4 mm, which would allow to resolve details finer than the innervation density.

A temporal code is observed for the perception of fine or micro features such as microdots and fine gratings. In a series of studies, Johansson and LaMotte (1983); LaMotte and Whitehouse (1986), and LaMotte and Srinivasan (1991) showed that humans can detect very small dots, on an otherwise smooth surface, of only 1 micron height with a diameter of ∼600 microns (3 microns with ∼230 microns diameter and 6 microns height with a diameter of ∼40 microns) and very fine textures (parallel bars 45 microns wide and spaced ∼100 microns) of only 0.1 microns height, when compared against a smooth surface in a 2AFC task. They found that lateral sliding is essential for these fine features to be perceived, as no sensitivity to the same set of stimuli was found with static touch. Neurophysiological recordings suggest that rapidly adapting mechanoreceptors (RA1 and RA2) have a primary role in the perception of the microdots and fine textures as they are sensitive to low amplitude and high-frequency vibrations (LaMotte and Srinivasan, 1991). The sliding movement is necessary to elicit skin vibrations (Manfredi et al., 2014) that in turn trigger the vibratory response of both types of rapidly adapting fibres.

### Open Questions

Several studies support the idea that coarse features (>0.1–0,2 mm) are mainly encoded in a spatial manner mediated by SA1 fibres, while fine feature (<0.1–0.2 mm) perception relies on the vibratory activity generated by stroking movement which mainly activates RA fibres (Blake et al., 1997; Hollins and Risner, 2000; Hollins et al., 2001). However, the prevalence of a spatial variation code over a temporal variation code might not be as evident in texture perception (e.g., Connor and Johnson, 1992), and the contribution of SA and RA may not be so distinct (Weber et al., 2013).

In fact, it is unlikely that the different mechanoreceptor types work in isolation. Instead tactile perception arises from the contribution of all the different units and their interaction (see Saal and Bensmaia, 2014). For example, Weber et al. (2013) showed that the perceived roughness of a wide range of textures is driven by three types of tactile units. On the one hand, coarser textures elicit the response of all afferent types but are best resolved from the spatiotemporal activation of SA1 fibres which is generated by the spatial layout of the texture in contact with the skin. On the other hand, finer features are mainly conveyed by the precise spike timing of RA and PC fibres (and to a less extent SA1 type). In a nutshell, Weber et al. (2013) showed that combining the response of RA1, RA2, and SA1 provides a more accurate prediction of the perceived texture than the response of a single unit type. Similarly, grip control can be achieved by a combination of the perceived object curvature, direction of motion (i.e., slip), onset or offset of contact, and applied pressure which are mediated by the four different classes of afferent fibres (Westling and Johansson, 1987; Birznieks et al., 2001; Jenmalm et al., 2003).

Furthermore, it is worth mentioning that although passive and active touch generate similar responses in the population of receptors and fibres, they differ in the extent to which the contact dynamics, and hence, the activation of tactile units can be controlled. Proprioceptive feedback and motor control allow exploratory patterns to be adjusted to optimise the sensory input (Kaim and Drewing, 2011), and can provide additional cues. For example, the applied force and speed alone can affect the signal in several ways. Higher force may result in greater contact area between the finger pad and the surface, generate stronger vibrations, and provide more reliable auditory cues when sliding.

Another question is how the state of the peripheral components affects the information encoded in the response of afferent fibres and transmitted to the central nervous system. As mentioned previously, skin and mechanoreceptors properties depend on the individual characteristics such as occupation, gender, age. People differ in terms of skin stiffness due, for instance, to the thickness of stratum corneum. Or, the afferent density which is inversely related to the finger pad area and decreases with ageing.

In order to extend the understanding of the relationship between neural activation and tactile perception, it becomes clear that models should account for the presence of all fibre types at population level, their properties, and a realistic definition of the skin. Yet, modelling the four afferent types together or each one at a time as well as the extent to which the modelled skin reproduces the complexity of the real skin requires a different range of skills and resources. Thus, in order to improve efficiency, the amount of details that are included in a model should be related to the type of stimuli simulated (e.g., static indentation vs. vibrations), type of skin contact (e.g., pressing vs. sliding, active vs. passive), and the task that is being analysed.

## Models of Tactile Neurons

The challenge of recording the population activity of the afferent fibres in combination with psychophysical testing, makes it difficult to understand how the information is encoded from behavioural or neurophysiological data alone. In order to further characterize the low-level mechanisms of tactile perception and to extend the knowledge of the underlying cognitive mechanisms, it is necessary to study the response of the four afferent populations working together, and the effects of skin properties on the neural activation. In this regard, computational modelling holds promise to bridge the gap between neurophysiological, psychophysical and neuroimaging studies. Although a model is built on the limited data from neurophysiology, it can provide further insights into how tactile features are extracted at population level and link the neural activation to perception. It allows examination of the recorded data from a different perspective and to make and test predictions faster by simply changing one or more parameters (e.g., skin stiffness and number of receptors). However, this approach carries the limitations embedded in the data on which it is built, such as the type of task used during the recordings.

### Introduction to Models

The most common approach to build and validate a model is to rely on neurophysiological data recorded on monkeys. Although they are devoid of SA2 fibres, monkeys’ tactile systems are very similar to the human tactile system (Parè et al., 2002). The literature on these types of studies provides more data when compared to those from recordings in humans but tends to be limited in the psychophysical tasks used.

**FIGURE 5:**
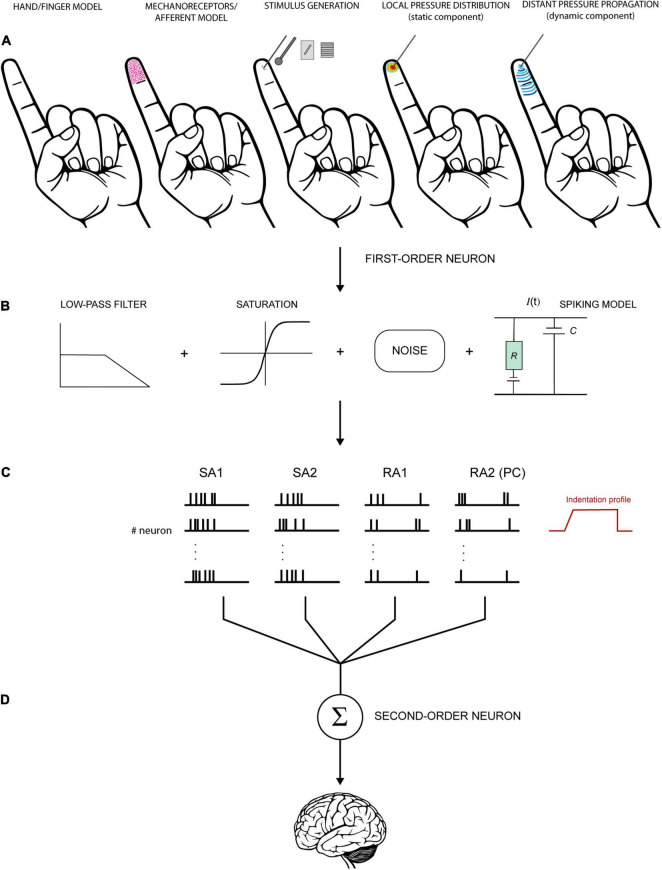
Schematic view of a hypothetical model to simulate the activity of tactile units of the hand. **(A)** First step involves the definition of the hand/finger model (shape, geometry, and mechanics), the depth and density of receptors/fibres, and the stimulus generation. Then, these factors are used to compute local and distant pressure distribution and derive the stresses acting at the receptors’ depth. **(B)** The mechanical output is fed into a neural model to generate spiking responses. The signal can be transformed through a series of functions to resemble the biological properties of the neurons. For example, low-pass filter is often used to account for the fact the tactile units do not respond above certain frequencies. Saturation reproduces the tendency of the neurons to saturate at high intensities. Eventually, noise is introduced to account for the random occurrence of the spikes in some conditions. **(C)** Spikes are generated for each receptor or each tactile unit (receptors and fibers together, first-order neurons). The activity of each receptor must be integrated to account for innervation branching observed in SA1 and RA1 but not in RA2 (PC) and SA2. **(D)** Subsequently, the output of first-order neurons is combined at the level of second-order neurons for further processing before is transmitted upstream. The last step is often overlooked in this kind of models but is fundamental to understand the nature of the information that is sent to the brain.

Generally, a computational model aimed at simulating the activity of afferent fibres comprises three major components: the stimulus (i.e., which stimuli can be simulated and how), the skin mechanics (i.e., how the finger is shaped and how the skin moves leading to mechanical stimulation of the receptors, mostly involving a continuum mechanics approach or a Finite Element Method of analysis), and the neural model (i.e., afferent type and response properties, how the neural dynamics are generated, usually on an integrate and fire basis). These components may be characterised from an engineering and mathematical perspective in terms of a stimulus transformation into a neural response by means of a series of functions and equations (Figure 5).

The goal of a model is to tractably implement the properties of each afferent type so as to produce an output when stimulated by a specific input. This normally includes the four types of fibres, and their properties, such as receptive field characteristics (e.g., small and large, single and multiple hotspots), firing rates, spike timing, frequency tuning, adaptation, edge enhancement, and surround suppression. In addition, the model should have a realistic definition of the finger shape and its mechanics (i.e., 3D shape, presence of hard structure like bones and nails, and viscoelasticity), with the possibility of simulating both static and sliding stimuli, and a faithful mechanotransduction process for a wide range of stimuli. In reality, all of this is not possible yet, mainly because of the computational demand and the lack of knowledge in some domains such as skin mechanics. Accordingly, all the models available focus on one or few aspects, come with limitations, and are far from exhaustive.

The next few paragraphs outline the main characteristics of a few selected models presented in Gerling et al. (2013); Saal et al. (2017), Hay and Pruszynski (2020), and Ouyang et al. (2021). The selection of these models was based on the fact that each successfully reproduces a small set of properties (e.g., slow versus fast adapting units and viscoelasticity of the skin) and is more or less suited for specific tasks as illustrated in the Application section. The models differ from one other with respect to the stimuli that are simulated, how they resolve skin mechanics and which afferents are included, the response properties of the simulated fibres, and their applications (for a summary see Table 1).

**TABLE 1:** Summary table of the main features of selected models.

Model	Gerling et al., 2013	Saal et al., 2017	Ouyang et al., 2021	Hay and Pruszynski, 2020
Data	Monkeys	Monkeys	Monkeys	Humans
Afferent population type	SA1	SA1, RA1, RA2	SA1, RA1, RA2	RA1, second-order neurons
Receptive field	Simple, no multiple hotspots	Simple, no multiple hotspots	Simple, no multiple hotspots	Complex with multiple hotspots
Response properties	Firing rate Spike timing Response adaptation	Firing rate Spike timing Frequency tuning Response adaptation Edge enhancement	Firing rate Spike timing Frequency tuning Response adaptation Edge enhancement	Firing rate Spike timing Response adaptation Edge enhancement
Models of skin mechanics	3D Finite Element Model resembling different layers and viscoelastic properties	Skin treated as a flat surface – continuum mechanics to derive deformation	Skin treated as a resistance network	No, skin is only represented by a grid as reference for receptors location
Neural dynamics	Leaky integrate and fire	Leaky integrate and fire	Leaky integrate and fire	No
Stimuli	Static indentation of cylinders, bars, and spheres	Static spatiotemporal indentation of single pins that can be combined to form complex shapes	Static spatiotemporal indentation of single pins that can be combined to form complex shapes	Static indentation of edges and dots
Applications	Predicting behavioural response from simulated neural response. Assessing the effects of realistic skin properties (e.g., viscoelasticity) on the skin response with static indentation of cylinders, bars, spheres. Evaluating potential mechanisms of peripheral sensory processing at the level of first-order neurons.	Predicting behavioural response from simulated neural response. Assessing the effects of finger properties (e.g., skin elasticity and afferent density) on the neural population response to static and vibratory stimuli having a wide range of shapes. Real-time generation of spike trains for robotics and neuroprosthetics. Evaluating potential mechanisms of peripheral sensory processing at the level of first-order neurons.	Predicting behavioural response from simulated neural response. Simulating the neural population response to static and vibratory stimuli having a wide range of shapes. Real-time generation of spike trains for robotics and neuroprosthetics. Evaluating potential mechanisms of peripheral sensory processing at the level of first-order neurons.	Predicting behavioural response from simulated neural response. Assessing the role of complex receptive fields on the neural population response to statically indented edges and dots. Evaluating potential mechanisms of peripheral sensory processing at the level of second-order neurons.
Code	n/a	bensmaialab.github.io/software/	github.com/ouyangqq	senselab.med.yale.edu/modeldb
Documentation	n/a	Yes	Limited	Limited

### Stimuli

Tactile inputs are of many different sorts as they depend on specific object characteristics, properties of the finger, and their interaction. Lederman and Klatzky (1987) identified two major classes of object properties: substance-related (i.e., roughness, slipperiness, hardness, and weight) and structure-related properties (i.e., weight, volume, and shape). In fact, real-world objects comprise a variety of these properties which, in combination with the different exploratory procedures that can be employed, generate a variety of possible tactile inputs. Accordingly, it would be difficult to replicate the full range of inputs that we deal with in real life, and researchers have to select the most relevant stimuli according to the scope of their model (Table 1).

Gerling et al. (2013) used cylinder, bar and sphere indenters of different but fixed size to validate their model of the finger and the resulting units’ response. These inputs were always simulated to be statically indented and no vibratory stimuli were considered. Saal et al. (2017) tried to extend the set of virtual stimuli by defining the input for their model as a single cylindrical pin or a set of pins that indent the skin orthogonally with spatiotemporal variations. Each pin is independent from the others with regards to location and indentation depth. As a result, it is possible to approximate different shapes from a single dot of a desired diameter to gratings, textures, curved lines, or other spatial patterns. In addition, their model allows the manipulation of the dynamics of the indentation including static stimuli with controlled onset and offset (i.e., ramp-and-hold) and vibratory stimuli (e.g., sinusoidal, diharmonic, etc.). Similarly, Ouyang et al. (2021) defined their stimulus as a single or a set of probes, with fixed diameter, indented orthogonally into the skin. Here, the probes can have different heights (i.e., indentation depth) that can be combined to produce many different spatial configurations. Their approach also allows direct creation of tactile input from a visual image. It is sufficient to extract height information from a grayscale 2D image and input it to the model. Similar to Saal et al. (2017), this model can be used to simulate static and vibratory stimuli but not sliding contacts.

Hay and Pruszynski (2020) built their model on neurophysiological data recorded in response to embossed dots and oriented bars sliding over the finger pad with specified velocity (Pruszynski and Johansson, 2014). The sliding movement was chosen to finely characterise the spatial layout of the recorded afferent’s receptive field and to investigate how the distribution of highly sensitive zones within each receptive field affects the neural response.

### Properties of the Virtual Skin

The implementation of skin mechanics and different types of afferents is subject to a trade-off between realism and computational efficiency. As a result, modelers have to make a choice about what elements to include and how to implement them (Table 1).

**FIGURE 6:**
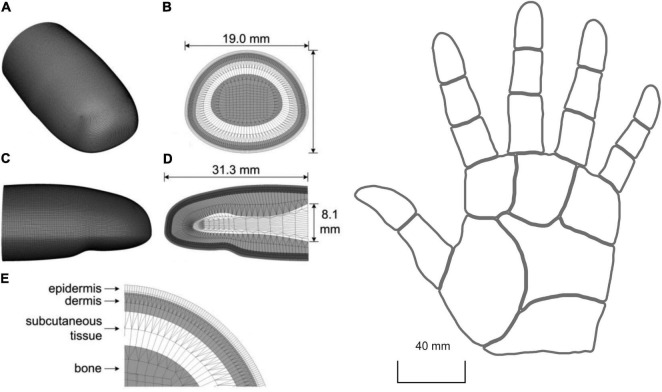
Left: 3D mesh of human distal phalange in Gerling et al. (2013). **(A)** overall mesh, **(B)** cross section of the mesh near the interconnect with the middle phalange, **(C,D)** longitudinal section for both the outer surface and inner mesh, and **(E)** four layers of microstructures. In **(E)** the epidermis is 0.471 mm thick (0.371 mm stratum corneum and 0.1 mm living epidermis) and the dermis is 1.153 mm thick. Right: 2D reconstruction of the virtual hand in Saal et al. (2017). Here, the skin is treated as a flat surface. (Left) republished with permission of The Institute of Electrical and Electronics Engineers, Incorporated (IEEE), from Gerling et al. (2013); permission conveyed through Copyright Clearance Center, Inc.

Gerling et al. (2013) used finite element modelling to create a 3D model of the human distal phalange consisting of about 276.000 elements and 232.000 nodes (Figure 6, left). They included the different layers of the skin and their properties such as viscoelasticity, but not anisotropy. Then, in order to derive the response of SA1 fibres, they used strain energy density as the input for their leaky integrate-and-fire neural model. This solution originated in the work of Phillips and Johnson (1981b) who found that SA1 firing rates closely correlate to maximum compressive strain and strain energy density generated in the skin. Subsequently, Sripati et al. (2006) developed the model of Phillips and Johnson (1981b) by implementing the RA1 fibres and testing how well different measures of stresses and strains can predict the neural response. They showed that maximum compressive strain and stress, maximum deformative strain and stress, maximum tensile strain, and relative change in receptor area are all good candidates to drive the response of both RA1 and SA1 afferent fibres.

Although computationally demanding, the realistic 3D shape and a subject-specific definition of the finger of a finite element model can provide a good understanding of the relationship between skin properties, neural activity, and tactile perception. In fact, individual differences in skin elasticity, finger size, and shape are likely to affect the skin mechanical response, the resulting contact area, pressure, friction, and mechanoreceptor activation. This is especially relevant in the presence of tangential loading, such as during object manipulation.

In contrast to finite element models, continuum mechanics simplifies the analysis allowing the skin response to be resolved more efficiently (Figure 6, right). In this approach, the skin is considered as a flat surface with homogenous elasticity, with isotropic and elastic behaviour, and devoid of any underlying hard structures. This method allows fast computation and produces characteristic responses in both slowly and rapidly adapting fibres. With this approach, Saal et al. (2017) were able to simulate the activity of multiple SA1, RA1, and RA2 fibres. This model was focused on the whole hand and allows the manipulation of the location and density of the units. Saal et al. (2017) exploited continuum mechanics to derive the stresses at the depth of receptor that are then used as input to a leaky integrate-and-fire neural model to generate trains of action potentials. The stresses are estimated for two different aspects of the indentation: a (quasi)static and a dynamic component. The former represents the resulting distribution of pressure over the skin close to the contact point, the latter accounts for the variations of pressure that propagates through the skin which cause the afferents to respond to vibrations far from the contact point.

Although simple, this approach is relatively cumbersome when dealing with complex stimuli because the resulting deformation is computed individually for each of the pins that form the stimulus. In order to increase the efficiency and reach real-time simulation of afferent response, Ouyang et al. (2021) went further to simplify the definition of the skin mechanics. The authors built the virtual skin as a resistance network made out of multiple connected nodes each representing a tactile unit. The units have fixed locations and are distributed only on the fingertip. Here, the assumption is that the actual pattern of indentation can be represented by a pattern of node voltages. The input currents for each node (i.e., afferent) are computed solely from the indentation depth of the stimulus image. The resulting voltages are processed by a two-channel filter in which low-pass and band pass filters mimic the static and dynamic aspect of the indentation. Then, an integrate and fire model is used to generate the action potentials. The advantage of this method is that the input currents are fast and easy to compute compared to the skin deformation and resulting stresses at receptor depth as in Gerling et al. (2013) ‘s or Saal et al. (2017)’s approaches. This makes this method more suitable for real-world applications such as neural prostheses and robotics where speed and accuracy may be crucial.

Importantly, all the skin models shown so far are limited to responses to stimuli indented orthogonally into the skin and do not include lateral sliding, the tangential forces, the friction between the skin and the stimulus nor the onset of slip which are relevant factors when simulating a sliding or a grasping movement.

A further approach is to omit any consideration of skin mechanics. Obviously, this prevents understanding of the link between skin properties and neural response, but it represents a viable solution to focus the efforts on the neural elements. For example, Hay and Pruszynski (2020) set up the virtual skin as a 12 mm × 12 mm grid uniquely designed for the arrangement of a set of modelled RA1 mechanoreceptors. They focused their modelling on an accurate definition of the relationship between mechanoreceptors, first-order neurons (i.e., afferent fibres), and second-order neurons (i.e., spinal cord and cuneate nucleus).

### Tactile Units of the Virtual Hand

Tactile neurons respond to stimuli in a specific manner. The response features include a receptive field which may be of varying size with single (SA2 and RA2) or multiple hotspots (SA1 and RA1), adaptation to constant stimuli, timing of individual action potentials, frequency tuning, and spatiotemporal sensitivity. For a model to be useful in aiding research about how tactile features are extracted, it is important that it is able to reproduce the response properties of interest (Table 1). For example, if we were to look at how vibratory stimuli are reflected in the spike timing, the model would have to simulate this feature accurately.

**FIGURE 7:**
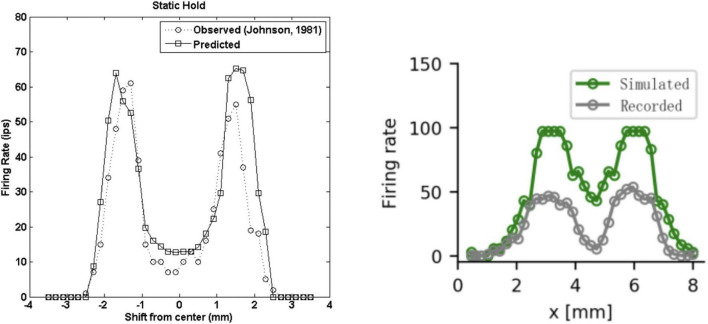
Firing rates during the static phase of the response to a 3 mm wide edge indented 1 mm. **(Left)** Recorded (dotted line, Phillips and Johnson, 1981a) and predicted (solid line, Gerling et al., 2013) SA1 firing rates as a function of the location with respect to the stimulus. Note the *x*-axis is different in the two graphs. **(Right)** Recorded (gray, Ouyang et al., 2021) and predicted (green, Phillips and Johnson, 1981a) SA1 firing rates as a function of the location with respect to the stimulus. Left image from Gerling et al. (2013). Right image from Ouyang et al. (2021). **(Left)** Republished with permission of The Institute of Electrical and Electronics Engineers, Incorporated (IEEE), from Ouyang et al. (2013); permission conveyed through Copyright Clearance Center, Inc. (2014). **(Left)** Republished with permission of The Institute of Electrical and Electronics Engineers, Incorporated (IEEE), from Gerling et al. (2013); permission conveyed through Copyright Clearance Center, Inc.

The finite element model of Gerling et al. (2013) focused on simulating a realistic skin response and the resulting activity of the population of simulated SA1 fibres. They showed that their model matches the proximal (<0.5 mm) and distal (0.5–5 mm) skin deflection observed in the work of Srinivasan (1989) in humans. Here, the skin deflection is simulated in response to a 50 microns line load and a 3.17 mm cylinder with 1 mm indentation depth, consisting of a dynamic ramp and a static hold. Interestingly, the spike timing and firing rate during the ramp and static phases, which closely matched the data on monkeys recorded by Phillips and Johnson (1981a), showed a faithful representation of the SA1 adaptation rate to statically indented stimuli. Although, this model does not implement vibratory stimuli, it outperforms the model of Ouyang et al. (2021) in reproducing the trend and the intensity of the response to indented stimuli (Figure 7) during the static phase of indentation.

A more systematic attempt to reproduce the response properties of tactile neurons has been made by Saal et al. (2017). Their model was fitted to data recorded from rhesus macaques (Muniak et al., 2007) when presented with sinusoidal and bandpass noise vibrations (frequency range 1–1,000 Hz) and tested with diharmonic stimuli of different frequency. Four main points emerge from their simulation.

First, the simulated firing rate and the spike timing correlated well with the actual data. The model can simulate the response of each afferent type to a static ramp-and-hold indentation that results in a different pattern of firing rate (i.e., adaptation, slow versus fast). In addition, the model is able to simulate the timing of spikes with a temporal precision from 3 to 8 ms for SA1 and RA1, and sub-millisecond precision for RA2. Although not entirely accurate, this range of values is acceptable as it has been shown that the stimulus information (i.e., vibratory frequency and texture) can be best decoded when spike trains are compared to one another with a temporal resolution of around 5 ms for RA1, 10 ms for SA1, and 2 ms for RA2 (Mackevicius et al., 2012; Weber et al., 2013). Second, the simulated receptive fields have similar features to the actual ones, including size, susceptibility to indentation depth (e.g., RA1 RFs increase with increasing indentation, but not SA1), and increased threshold amplitude with increasing distance from the RF centre. However, the innervation pattern, and hence, the receptive field shape does not match the real characteristics. In the human hand, SA1 and RA1 are connected to multiple receptor organs and each receptor is innervated by multiple fibres. Instead, the mapping between the modelled receptors and fibres is 1 to 1. Accordingly, one must keep in mind that the nature of these virtual receptive fields might affect the activation of the simulated tactile fibres. Third, tactile neurons are sensitive to different frequency ranges. Here, the simulated response of each class of fibre to sinusoidal vibration mirrors the actual sensitivity profile with respect to the minimum amplitude to elicit a single spike (i.e., absolute threshold), and the minimum amplitude to generate at least one spike per cycle (i.e., tuning threshold). Fourth, the simulated SA1 and RA1 fibres reflect the spatial layout of the applied stimulus which is achieved by a combination of edge enhancement and surround suppression, which is more evident for SA1.

The work of Ouyang et al. (2021) has a number of similarities with Saal et al. (2017). The authors used the same dataset as in Saal et al. (2017) for fitting their model (Muniak et al., 2007), and despite a different solution to reproduce the interaction between the skin and the stimulus, they obtained similar results with regards to the precision of the firing rate and spike timing when compared to real data. In particular, the simulated firing rates reproduce the trend observed when a probe with a diameter of 1 mm is indented with different frequencies and depths. The rate increases with depth as expected, although the number of spikes does not match the actual measurements perfectly. On the other hand, spike timing has a temporal precision slightly higher than in Saal et al. (2017) for SA1 and RA2 (3–6 ms versus 3–8 ms), and similar for RA2. Using a resistance network to model the skin mechanics proves an interesting solution when dealing with receptive field characteristics. This model provides a viable way to characterise the receptive field size and the changes that occur in response to different indentation depths. Also, it can generate the characteristic edge enhancement and surround suppression, prominent in SA1 and less in RA1, observed in response to statically indented texture, form and shape.

**FIGURE 8:**
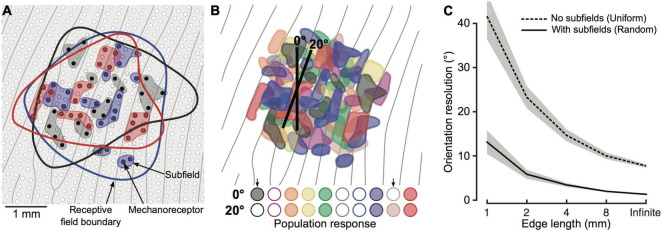
**(A)** Schematic of skin patch with papillary ridges (grey lines) and mechanoreceptors (white and colored dots). Blue, black and red dots represent receptors innervated by one of three first-order tactile neurons. Colored contour represents first-order neurons receptive field, while shaded area behind the colored dot represents subfields. **(B)** Color-coded subfields for 10 first-order tactile neurons. Representation of 10 first-order tactile neurons with overlapping receptive field and subfields (color-coded). First-order neurons are activated if the edge falls on one subfield. Here, the activation response is shown for 10 neurons and 2 edges of 2 mm with different orientation (0° and 20°). Colored circles are filled if the neurons is active and empty otherwise. **(C)** Output of the two tested models (subfields vs. uniform sensitivity). The lines indicate the mean and the shaded areas represent the 95% confidence interval. Image from Pruszynski et al. (2018) reproduced under the terms of a Creative Commons Attribution License.

It is worth noting that none of the above models included a realistic definition of the innervation branching that characterises SA1 and RA1 units. In this regard, Pruszynski et al. (2018) proposed a model that takes into account the complexity of the receptive field of first-order tactile neurons having multiple subfields. They compare this model to a second version in which all units had uniform sensitivity in the context of edge orientation discrimination. In both versions, the first-order neurons have the same receptive field size but are connected to either a single receptor that covers the entire area for the simple model or multiple receptors that form random subfields of sensitivity (Figure 8A). The activation of each first-order neuron in the two models can only be either 0 or 1 based on whether the virtual edge falls on the receptor area. As a result, the population response of complex receptive fields shows more variability (Figure 8B) and can better account for the behavioural results (Figure 8C).

Recently, Hay and Pruszynski (2020) extended these findings with a model built on data from microneurography recordings in humans presented with embossed dots and bars of different orientations (Pruszynski and Johansson, 2014). Here, the individual RA1 afferents are designed to be connected to multiple mechanoreceptors arranged on a 12 mm × 12 mm grid and spaced 0.1 mm. The location and the weight (i.e., output) of the mechanoreceptors as well as the maximal firing rate of the first-order neuron were free parameters determined by a genetic algorithm. Locations were searched within the area of responsivity of the recorded neurons and different numbers of innervated mechanoreceptors were used for optimisation runs. The best fit model was obtained at around 20 mechanoreceptors, each having different weights representing the different degree of sensitivity within the same receptive field, in line with empirical findings (Nolano et al., 2003). Also, this model predicted the spike timing and firing rate with high accuracy in response to edges with different orientation. In addition, the authors included a model for input integration at the level of second-order neurons and tested whether the output can discriminate between different oriented stimuli. First, they simulated the response of 330 RA1 units and convolved it with two post-synaptic waveforms having short or long decay representing two types of synapse, AMPA and NMDA, respectively. Then, the weighted sum of the convolved outputs is computed to simulate the integration of the signal mediated by second-order neurons.

### Applications

One of the main goals of modelling the activity of tactile afferent fibres is to clarify the nature of the inputs the central nervous system receives and to help explain how individual differences (e.g., skin properties in the ageing population) contribute to shape the tactile signals at early stages. In this review, the focus has been on how modelling can be employed to better understand the basic sensory mechanisms that underlie and enable tactile perception, and how it can be used in combination with behavioural experimentation. In this section we highlight the applicability of each selected model.

Finite element 2D and 3D models can be built with different level of detail, including macrostructures (e.g., finger shape and skin layers) as well as microstructures (e.g., fingerprint, dermal papillae, etc.). Although the main strength of finite element modelling is the realistic definition of the finger properties to accurately estimate the deformation of the skin and the stresses acting on the mechanoreceptors, it can certainly be used in combination with psychophysics to assess potential mechanisms of peripheral sensory processing. For example, Gerling et al. (2013) showed that the simulated firing rate in response to spherical indenters with different radii can predict the behavioural performance in a previous psychophysical experiment on curvature discrimination (i.e., Goodwin et al., 1991). Interestingly, they tested two potential encoding strategies based on first spike latencies and the firing rate in the dynamic and static hold, separately. These two coding schemes are Gradient Sum Method and Euclidean Distance Method (see Gerling et al., 2013 for details). They found that the firing rate during the static phase of indentation produced a better fit to the behavioural data for both methods. Notwithstanding this finding, the first spike latencies and the firing rate in the dynamic phase were still good predictors suggesting that these two measures may carry information about the stimulus as early as the initial phase of the stimulation. However, this model does not include RA1 fibres, which have good spatial resolution, and may contribute to the encoding of stimulus information. Also, only static stimuli are included while vibrations and dynamic contacts are not.

Continuum mechanics models can provide further insights into the mechanisms underlying tactile perception and offer a more efficient way to simulate the spiking response. A good example of this is a study of Delhaye et al. (2019) on edge orientation. They used the model of Saal et al. (2017) to simulate the activity of the entire population of SA1 and RA1 fibres of the finger pad in response to indented edges with different orientation. They sought to determine how the information about the geometric feature (e.g., orientation) in contact with the skin can be extracted so efficiently as to enable rapid object manipulation. Although previous studies show that shape can be extracted from the spatial variation of the response of the tactile fibres (e.g., Phillips and Johnson, 1981a), the focus was on the mean firing rates over long time intervals and only for a few recorded afferents. As a result, it is not clear whether such a spatial code can be accurate and fast enough. Delhaye et al. (2019) used a classification approach to show that the spatial pattern of activation of simulated SA1 and RA1 fibres contains accurate information about edge orientation and that it can be decoded starting from the early phase of the indentation. In particular, they found that edge orientation can be decoded within 10 ms, when most afferents have produced only a single spike, with an error of 5°, and within 50 ms with an error of 1° to 3°. In addition, they found that taking spike timing into account did not improve performance. These results suggest that a spatial variation code is a better candidate for how peripheral neurons encode geometric features. This work has some limitations too. For example, the model output does not faithfully reproduce the trial-to-trial variability observed in real neurons which may boost the orientation decoding performance. The authors tried to overcome this issue by jittering the stimulus position on each trial to resemble this variability. Most importantly, the model does not provide an accurate picture of neuronal receptive fields. Real SA1 and FA1 neurons have multiple hotspots of sensitivity, not implemented in Saal’s model, which may affect how a stimulus activates the population of afferents.

The role of the complex structure of receptive fields is highlighted in the results of Hay and Pruszynski (2020). Their model of RA1 units shows that such complexity enables the discrimination of fine orientations (e.g., −1° vs. +1°) under different level of stimulus noise and outperforms a similar model with uniform receptive fields. In addition, they implemented the population response of second-order neurons, connected to first-order neurons with both AMPA- and NMDA-like connections. Using different stimulus presentation time windows (from 5 ms to unlimited time) revealed that AMPA- and NMDA-like synapses are more robust to noise within short and long-time windows, respectively. These results suggest that AMPA-like connections may allow the computations that underlie fast object manipulations while NMDA-like connections may be involved in object discrimination. Overall, this work supports the possibility of peripheral sensory processing of geometric features as opposed to the traditional view of central processing (Bensmaia et al., 2008b) and can be used to assess similar questions related to shape or texture. Similar to other models, these results provide only a partial picture due to the lack of SA1 neurons which are known to have very fine spatial resolution. In addition, Hay and Pruszynski (2020) implemented sliding stimuli with no consideration of skin mechanics. The sliding movement produces a complex mechanical response that may result in a rearrangement of the receptive fields depending on scanning direction (Jarocka et al., 2021).

The model of Saal et al. (2017) has also been used to investigate the activity of RA2 units in contexts where vibrations are the only source of tactile information. Miller et al. (2019) simulated the response of RA2 fibers to corroborate their neurophysiological findings in a tool sensing task. First, the authors showed that the primary somatosensory cortex can rapidly and efficiently access the information relative to where a rod, hold in the hand, is hit (close vs. far). This is reflected by a repetition suppression effect in the somatosensory evoked potential between 44 and 108 ms. This finding suggests that mapping touch on an external tool is achieved in a manner similar to somatosensation in terms of temporal dynamics and brain area involved. Miller et al. (2019) then used the experimental acceleration recordings, collected by hitting the rod at close or far locations, as the signal to stimulate the virtual RA2. They showed that the simulated spiking patterns carry information about location as early as 20 ms, a time-course compatible with their neurophysiological findings.

As an additional example we suggest how Saal et al. (2017) model can be used to investigate the impact of skin elasticity and afferent density on the encoding of the stimulus. It is known that these factors change with ageing (Yang et al., 2018; García-Piqueras et al., 2019) which is also characterised by a deterioration of tactile spatial sensitivity. However, there is no evidence to support a link between these age-related anatomical and morphological changes and poorer performance. To address this question, Saal’s model can be used to simulate the neural activity for young and elderly group in response to 2-point discrimination task and estimate the perceptual performance based on the virtual response. This model allows the two groups to be defined by setting lower elasticity and lower afferent density for the elderly and generate the virtual response to a single pin and two-pins at different separation levels for each group. The model output can then be used as input for an LDA classifier trained to discriminate between the single pin and the two-pins at each separation level, separately, and estimate the perceptual thresholds by fitting a logistic function to the classifier output.

In summary, modelling can be used to investigate the sensory mechanisms of tactile perception including potential coding strategies and the extent to which each afferent type contributes to the encoding of the stimulus, and to assess the effects of skin properties.

## Discussion

Somatosensory processing begins at the periphery with the transformation of the mechanical stimulation into neural activity. The components involved in processing tactile signals enable the multifaceted aspects of touch, which include object and body perception, social and affective interaction, and they provide the basis for action control. Tactile information is transmitted from the skin to the upper spinal cord, and on to the thalamus, the primary and secondary somatosensory areas, which are the ending point of a hierarchical organisation with various overlapping networks involved in different functions (de Haan and Dijkerman, 2020).

Simulating the activity of tactile neurons and estimating the information conveyed by their activation pattern under different circumstances is crucial to assess the mechanisms acting at lower levels of the somatosensory system and to predict the impact of different skin and peripheral neuron properties.

The state of the art of this type of models has enabled research to focus on the basic sensory mechanisms underlying tactile perception without the need for challenging microneurography recordings. Research in different fields can benefit from this approach as it can provide additional evidence to test experimental hypotheses. However, it is important to be aware of the limitations of the model being used and to interpret results with caution. Models are built on real data recorded from a limited number of tactile fibres when stimulated with specific stimuli (e.g., vibration, edge indentation, etc.), and only some of the properties of the skin and neurons can be currently reproduced. Although these limitations prevent complete reliance on modelling for hypothesis testing, this approach can provide useful insight into open questions that cannot still be addressed with microneurography. For example, it can help address the effects of contact dynamics and the state of the peripheral sensory components on the information subserving tactile perception.

Interestingly, Saal et al. (2017)’s model allows some factors to be manipulated with ease. It is possible to change the density and distribution of the simulated fibres, the stiffness of the skin, the position of the indentation, or its depth on each trial. Having the possibility to manipulate these properties may help address questions related to the decay in spatial sensitivity observed in the elderly, a group which typically has stiffer skin and fewer mechanoreceptors, as described in the previous sections. One advantage of this model over the others is its ease of use. The available bundle of functions for MATLAB and Python, supported with usage examples, makes it accessible even to users that are not familiar with modelling (for code and documentation availability of all models see Table 1). Hay and Pruszynski (2020) and Ouyang et al. (2021) also provided the code for their model but only very limited documentation to help the users running the scripts. Gerling et al. (2013), instead, did not release their code but explained the framework in detail on their original publication.

Another limitation of all the models presented in this review is that none of them implement skin mechanics and neural dynamics in the presence of tangential loading. In fact, an important aspect of tactile perception is the dynamic behaviour of the skin during object manipulation and sliding movement in which normal and tangential forces act concurrently. In these contexts, the friction between the finger and the surfaces well as the 3D geometry of the finger are extremely important. The development of a realistic 3D definition of the finger geometry will be crucial to understand the effects of tangential loading on the bulk and local deformation of the skin, and hence on the resulting neural response.

Finally, the models discussed in this review cannot directly account for whether and how the information coming from peripheral neurons is used at higher level. For example, the fact that two different stimuli are discriminable based on a spatial code does not imply that the brain can directly extract this information. Similarly, faster sliding movement on the same texture will produce higher frequency vibrations in the skin and higher firing rates in the peripheral neurons (Greenspon et al., 2020) posing the problem of understanding how perceptual constancy is achieved for the same tactile stimulus under different conditions. In this regard, a recent study on intracortical recordings of rhesus macaques S1 area by Lieber and Bensmaia (2020) provides some insights. The authors showed that scanning the same texture at different speeds generates higher variability in the afferent fibers compared to the cortical neurons in S1. These results suggest that perceptual constancy for the same texture explored at different speed stem from the property of S1 neurons which can represent texture and speed in a relatively independent fashion.

Accordingly, further models are needed to establish how the cortical representations are formed, maintained and reorganized (e.g., Detorakis and Rougier, 2012), and perceptual judgment are made by taking into account additional factors such as memory decay and perceptual noise (e.g., Metzger and Drewing, 2021).

Future work should be aimed at improving the understanding of the dynamic behaviour of the skin and in general of its mechanics as well as of the mechanotransduction process and the individual differences. The goal is to develop models that implement realistic 3D finger geometry, skin and mechanoreceptors properties, includes SA2 fibres, and is able to reproduce the dynamic aspect of touch. Improving the accuracy, generalizability and efficiency of these models will help research in other related fields such as cognitive neuroscience, psychology, and neurorehabilitation.

## Author Contributions

DD conceptualised the review and drafted the manuscript. DD, MD, and AW critically revised and approved the manuscript for submission. All authors contributed to the article and approved the submitted version.

## Conflict of Interest

The authors declare that the research was conducted in the absence of any commercial or financial relationships that could be construed as a potential conflict of interest.

## Publisher’s Note

All claims expressed in this article are solely those of the authors and do not necessarily represent those of their affiliated organizations, or those of the publisher, the editors and the reviewers. Any product that may be evaluated in this article, or claim that may be made by its manufacturer, is not guaranteed or endorsed by the publisher.
